# Supplementary material for: Molecular Characterization of Odorant-Binding Protein Genes Associated with Host-Seeking Behavior in Oides leucomelaena
Source: Int J Mol Sci. 2024 Aug 30;25(17):9436. doi: 10.3390/ijms25179436 (PMC11394801; doi:10.3390/ijms25179436)
Supplement: Supplementary file 1 [file ijms-25-09436-s001.zip › Figure S1B.pdf]

```
              1       10                        20
OleuOBP11   ..MLKMKLLYIP................LVFLFLAVVS............AAPTK
OleuOBP28   .....MNLLYIP................LVLLFLAAVS............AAPTK
OleuOBP22   MLLIYRWLGRS....EENISVPFRLGADKGLSRLSECKWKWTCDYL....TELGE
OleuOBP31   MINIERNRNVL....NEFI........EQLLQHCYFQKYGATADTS....QQLGE
OleuOBP24   ...MKNEIIILFIFY.............TLSVHGFLQK............RDYGP
OleuOBP25   ...MKYEIIILSIFH.............TLSVHGVLQK............RDYGP
OleuOBP35   .....MKFLYVT...............LVVCLFVAVVS............AAPTK
OleuOBP14   .....MRSLYIF................VLLLALVLVS............GAPTN
OleuOBP27   ...MKLAVVLLLVVA............FTNDAYGYLEY............KDFGP
OleuOBP30   ...MKLVILLVCLYS.............GNAVYGYVQD............ADLAP
OleuOBP36   ....................................................MSP
OleuOBP32   .....MKHLIIF.................VLTFASIHC..............FTE
OleuOBP38   .......................................................
OleuOBP21   ....MKISLTIY................LITCATAVY.............ANTRR
OleuOBP42   ...MKSIIALALC...............LTVVNCAMDA............ALFSP
OleuOBP16   .....MKFLIVF................LVFAICSVN.............AEITP
OleuOBP20   .....MKFVIVF................MVFCVCYVN.............AALLP
OleuOBP29   .....MKYLIIVC...............FVAVVPTEI.............KALME
OleuOBP26   .......................................................
OleuOBP23   .....MKTACVFLC..............IVVAALAYNF............PYLPE
OleuOBP41   .......................................................
OleuOBP34   MLINSIKWIVFFVC..............IQMARCALDF............KEYNE
OleuOBP12   MYYLILIYTILITLT.............FLKAAPSINMS...........SAEMA
OleuOBP33   ....MLSTSKMFS.............AVILIVVCSIIGAF..........SETPA
OleuOBP40   ...MKFEVSIAQVMWKKEK........LLVRSINSFQGPKIQANMALFAILAMNS
OleuOBP1    .......................................................
OleuOBP15   .....................................................ML
```

```
                30        40        50        60        70
OleuOBP11  EEQEKIKKIHEECQSDPNTHID..DAVLEKLRKG.EKVDEP.NLGKHALCMN..V
OleuOBP28  EEQEESLKIHEECQSDPSTHID..DKVLQKLRKG.EFVDEP.NLAKHALCLN..V
OleuOBP22  HLEKDFKIWHEKCI.TLFPTVT..DKDITDMRRG.KFNDTNTELKKYNACIY..L
OleuOBP31  PLEKDFKMWHEQCT.TLFPTVT..NNDITDMRRG.KFNDTNTELKKYNACIY..L
OleuOBP24  GLHGIVDVLHNSC..KFLSGTT..ENLIDQLKDG.KFPEDD.SLKRYLYCLYTIK
OleuOBP25  GLLGIVDIHHNSC..KFLTGTT..ENLIDQLKDG.NFPEDD.SLKRYVYCLYTIK
OleuOBP35  EDWVELQKIHQACQQDPNTRID..ETIMEKIQNG.LRITEP.NLPKHTLCMH..V
OleuOBP14  KAKELIDRVHDECQANPETHID..DATVEKALKG.EHVDDP.KLPKHALCMN..V
OleuOBP27  TLLKEIEEIHQFC..VLFTGTT..EELIDGVRNG.RFPDDQPSIQKYTLCIW..R
OleuOBP30  NMLEGLKEWSYFC..KLFTGAP..PELISNIRSG.TYTGNDISVKRYNLCLW..K
OleuOBP36  SLLAGLKEWNYFC..KIFTDAP..QNLIDSVRSG.TYTGDDLSVKKYNLCIW..K
OleuOBP32  SYNEKLKKIVKECQDNEATRVP..VEWLDANQAV.VTSHIE.EAGPHILCRL..K
OleuOBP38  .....MDKVIEDCQADPNTRAS..DEWIKTED....LETAD.KEGAHILCRL..T
OleuOBP21  APNLKYQDARKKCQDDPATSLD..RETLKNAFRG...DVDAVVFGAHALCIY..K
OleuOBP42  KLAKLAKQLRIAC..KSRSGCD..EKFIDKLKVA.DFSNPDPTVKVYLNCLW..D
OleuOBP16  ELKEKLVAHTAEC..LKQSGVS..TELVQKSREG.QYAEDP.KLKDYFFCVT..K
OleuOBP20  EQKEKLSAYNTEC..MKQSGVN..PEIVQQARQG.QYSEDQ.KLKDHIFCVS..K
OleuOBP29  KEHAKLMALHEKC..LPTSGAT..TEMVQRAMAG.DFPEDP.EFKQHIYCIS..Q
OleuOBP26  ....MFAEIHRECQADPHTYCD..EDKLRNLHNN...IHDK.QTGIHMNCMA..E
OleuOBP23  NERIRFADIHRECQADPHTYCD..EDKLRNLHNN...IHDK.QTGIHMNCMA..E
OleuOBP41  ....MLKLTHDECQSDPETYCD..EDKLRNLDDN...IDDK.QVGNHMLCMA..I
OleuOBP34  NLQRDSVKWSAKC..KILTKFE..DKWLSEAKKG.NIMD...EIGDYYMCLW..L
OleuOBP12  ADMKLREKMGLEC..LKQVKLD..KKIVDKAVATLEFIEDK.KYKEFLACSY..K
OleuOBP33  EAHKRMQDTYIECG.KSSHYTE..QTFRKDIRNG...VQTD.DLKNFNYCIF..D
OleuOBP40  TSKEKFVITQPKLLEGVANCSEKFGIGIPTLFEGLQKNNQDPKLKEYNYCII..N
OleuOBP1   ............................................MAQMACID...
OleuOBP15  CVFQCVGQKNDIIADNGDLQADKFEKYIKEKLAG.FDYLVPVQDEIIKGCMQ..D
```

```
                80        90         100       110           120
OleuOBP11   KAGVQKENGDIIVDVLRSKLEQHV.DKDKVDKIIEKCG...TRKSGTTA.EEAAI
OleuOBP28   KHGVQTRNGDVIVDELRSKLEQFI.DKDNVDKVIQKCG...TRNANITA.EEAAI
OleuOBP22   INKGINPDMTVDKEVLEYYLPPGL.DEDQYLGYLDCVTK.SRNEGPEVSVVEKYW
OleuOBP31   ISKSVSPDMIINKEVLEYYYVPGSFDEDEYIGFMDCVKK.SRDEGPEVTIIEKYW
OleuOBP24   EGLVLNENMEYIIDKLLYYLPDMH..QEDFGFYTGCNQQ.EMRSVSERPIIESIW
OleuOBP25   RSVVLDENMQFSIDKLAYYVPDMH..QDDFGYYVGCSKQ.EVKNVSERSMIESIW
OleuOBP35   KGGLQSENGDIIADKLKIILGKVIDDTTKLDEAIEKCS...TKEAGITA.EDFAV
OleuOBP14   KLGIQNENGDVNVDKLRDGIGKRTEDTDKVDKLIKKCG...TRTPGNTP.GEAAI
OleuOBP27   GAGELDRNYRIIESKLRSYLTDLN..SQDAEVYLQCNDS.ALKLPIQDP.VKKLW
OleuOBP30   ISGALDRNLNLNIDQLRAYQTPST..LSNVDIYAKCLTH.ALKLGKADD.VEKIW
OleuOBP36   ISESLDSNLKINSDLLKKFETPLT..IGNVPIYEKCNKQ.ALELATDCP.VEKIW
OleuOBP32   KVGSFKENGDIDKNAMKQDLLSAIDNADKVKKIMKKCT...KHKRNWTP.EKNAI
OleuOBP38   KIGSLKPNGDIDKEAMENDLKKGIDDTEIVQMIVKKCG...TRKPSTTA.EESAV
OleuOBP21   HLGILNNHAEINKTELRYSLNYVITESDKLEEAVQFCS.....VKKATP.EETAV
OleuOBP42   ESKVVNSNLDIDKDLLYDLIPTTL.RDEMYDIYVRCIEE.SKKIENLDY.VEHTW
OleuOBP16   KFGILNDAGDIQRDVLKNQITTVFGDEALAERSIAECA.....VKQASG.LETAY
OleuOBP20   KIGLQNEAGDIQRDVLKQKFSAALGDEAAAEKLIALCA.....VKRENG.PETAY
OleuOBP29   GMGFFKENGEIDMDVVESELNSRITDPAVREEVKTKCL.....VMKETP.QDTAL
OleuOBP26   KAGLMN.HGKLDIETIRKKISLVV.HPSKVDSVVHNCC.....KEGENA.GVTAN
OleuOBP23   KAGLMN.HGKLDIETIRKKI...................................
OleuOBP41   KIGLMRANGEIKLIALQHIISLIITTEARASKLVSICD.....QRAESP.RKTAV
OleuOBP34   FSDALDLNFVIDHEKLDYYLPDLLKSKEAAEY..AKCGE.VQRKEDKSF.KERIV
OleuOBP12   KQGYQDLNGNILYQNIIEFLKRFYNISDL..KSVEQCKH.TSASGSDH..GELAF
OleuOBP33   KIGLLKKDREIDFDLVEKFAKAFV..PKKVEEVMTNCA.....KTKRTP.KETAL
OleuOBP40   YVGYLNGN.VYNEKLTREHTEQQI..PLLAEEFLKTCM.....VVKDTP.QETAY
OleuOBP1    ......DDGIIDEEATVAVLPEEY..REKVGPVIKKCG....TKKGKTP.CENAW
OleuOBP15   VKNTTQSNTKCKPDGLAVDFFLF......KNIQLNCPD.DQIKDKQFCQKLQERL
```

```
               130       140
                 .         .
OleuOBP11   EFDKCIRGPPPPRDGHHGGPPPHH...............................
OleuOBP28   ELDKCIRG...TRDEHHDDPALHH...............................
OleuOBP22   QITKCDYK.........RSPQEFLWD.............................
OleuOBP31   QITKCHYK.........ESPKDFIWD.............................
OleuOBP24   NMQKCIQK.......TVDAPHYIVF..............................
OleuOBP25   NMQKCIQS.......KVDAPVSIFFCFNPFSGKYLVVKLYRHKSI..........
OleuOBP35   HLASCIRG....YRLHTGPPPPHH...............................
OleuOBP14   ELSKCLHE.......FGHHGHGHH...............................
OleuOBP27   EMQKCIHR.......TIPKERYIFF..............................
OleuOBP30   EMQQCIQK.......NVPPEKLIYY..............................
OleuOBP36   QMQKCIQE.......HVQPEKLIYYVHASLLSTRHPRLGHTPLVRRFPSPVCLSG
OleuOBP32   KNYSCIMK........FSS....................................
OleuOBP38   ENFRCVLS.......TFNTLSQ.................................
OleuOBP21   AIFKCTKK........AMAPYLPYSDM............................
OleuOBP42   GIAQCIYK........CKPAQWLMF..............................
OleuOBP16   HIVKCFKE.........KSPQHASLV.............................
OleuOBP20   QSMKCYKE.........NSPQKITLT.............................
OleuOBP29   DSTKCLFT..........FKDLIN...............................
OleuOBP26   SLFVCFVK........NDAPYYHKL..............................
OleuOBP23   .......................................................
OleuOBP41   FLWKCFID........HGIPYYHKL..............................
OleuOBP34   GLGKCLYG........INPEKFIIF..............................
OleuOBP12   NAMKCVIKN......LQMIEGESSFEDNNI.........................
OleuOBP33   KFTRCASK........IVFEY..................................
OleuOBP40   YLSKCLTT.............FVHL..............................
OleuOBP1    LTNICYQS........EALDDYFLI..............................
OleuOBP15   KQGRDLFG.........PPPPPPEN..............................
```

**OleuOBP11**	....
**OleuOBP28**	....
**OleuOBP22**	....
**OleuOBP31**	....
**OleuOBP24**	....
**OleuOBP25**	....
**OleuOBP35**	....
**OleuOBP14**	....
**OleuOBP27**	....
**OleuOBP30**	....
**OleuOBP36**	RTMGLCRCSRRSPNRPFRRFRTHTGRLLPRLHSIAIRHTLLRLGATPSPTMPSHP
**OleuOBP32**	....
**OleuOBP38**	....
**OleuOBP21**	....
**OleuOBP42**	....
**OleuOBP16**	....
**OleuOBP20**	....
**OleuOBP29**	....
**OleuOBP26**	....
**OleuOBP23**	....
**OleuOBP41**	....
**OleuOBP34**	....
**OleuOBP12**	....
**OleuOBP33**	....
**OleuOBP40**	....
**OleuOBP1**	....
**OleuOBP15**	....

**OleuOBP11**	....
**OleuOBP28**	....
**OleuOBP22**	....
**OleuOBP31**	....
**OleuOBP24**	....
**OleuOBP25**	....
**OleuOBP35**	....
**OleuOBP14**	....
**OleuOBP27**	....
**OleuOBP30**	....
**OleuOBP36**	SVCVQLLCAISGAG
**OleuOBP32**	....
**OleuOBP38**	....
**OleuOBP21**	....
**OleuOBP42**	....
**OleuOBP16**	....
**OleuOBP20**	....
**OleuOBP29**	....
**OleuOBP26**	....
**OleuOBP23**	....
**OleuOBP41**	....
**OleuOBP34**	....
**OleuOBP12**	....
**OleuOBP33**	....
**OleuOBP40**	....
**OleuOBP1**	....
**OleuOBP15**	....
