# Supplementary figures and images for: Molecular Characterization of Odorant-Binding Protein Genes Associated with Host-Seeking Behavior in Oides leucomelaena
Source: Int J Mol Sci. 2024 Aug 30;25(17):9436. doi: 10.3390/ijms25179436 (PMC11394801; doi:10.3390/ijms25179436)

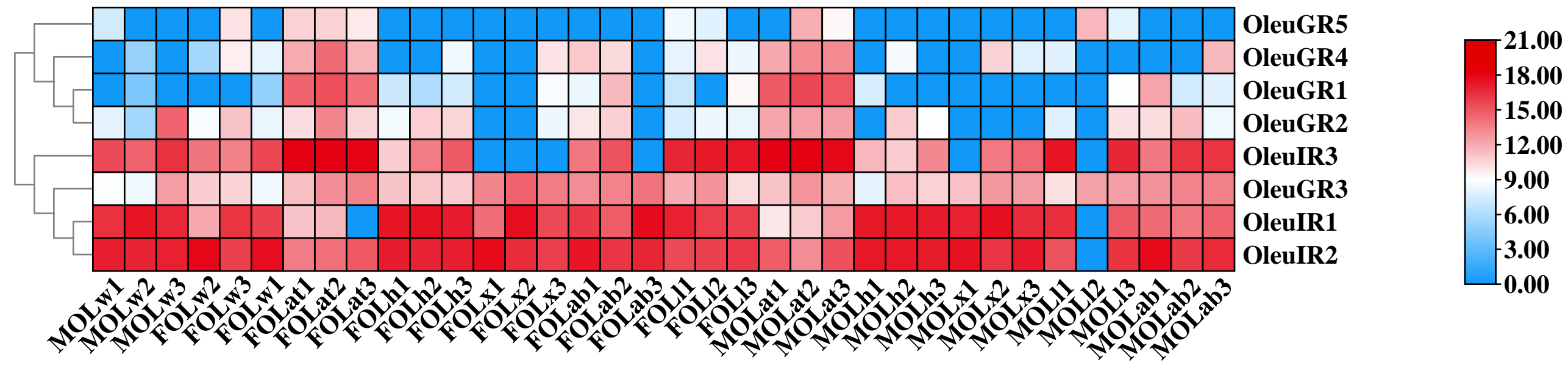

Supplement: Supplementary file 1 [file ijms-25-09436-s001.zip › Figure S1A.pdf]
